# Learning, exploitation and bias in games

**DOI:** 10.1371/journal.pone.0246588

**Published:** 2021-02-05

**Authors:** John M. McNamara, Alasdair I. Houston, Olof Leimar

**Affiliations:** 1 School of Mathematics, University of Bristol, Bristol, United Kingdom; 2 School of Biological Sciences, University of Bristol, Bristol, United Kingdom; 3 Department of Zoology, Stockholm University, Stockholm, Sweden; University of Electronic Science and Technology of China, CHINA

## Abstract

We focus on learning during development in a group of individuals that play a competitive game with each other. The game has two actions and there is negative frequency dependence. We define the distribution of actions by group members to be an equilibrium configuration if no individual can improve its payoff by unilaterally changing its action. We show that at this equilibrium, one action is preferred in the sense that those taking the preferred action have a higher payoff than those taking the other, more prosocial, action. We explore the consequences of a simple ‘unbiased’ reinforcement learning rule during development, showing that groups reach an approximate equilibrium distribution, so that some achieve a higher payoff than others. Because there is learning, an individual’s behaviour can influence the future behaviour of others. We show that, as a consequence, there is the potential for an individual to exploit others by influencing them to be the ones to take the non-preferred action. Using an evolutionary simulation, we show that population members can avoid being exploited by over-valuing rewards obtained from the preferred option during learning, an example of a bias that is ‘rational’.

## Introduction

In this paper we are concerned with a group that stays together for some time, perhaps during development, with group members competing with each other for a resource such as food. We idealise this situation by assuming that competition leads to each contestant playing a series of rounds of a focal game, focussing on how the structure of this game affects learning within the group.

When members of a group compete for resources, some actions are typically more prosocial than others; some actions benefit others, other actions are deleterious to others. For example in the classic Producer-Scrounger game [1, 2], producers, who search for food, benefit scroungers, who exploit the food that has been found by others. In contrast, scroungers reduce the food found by other group members. If group members play the Hawk-Dove game in pairwise contests, then the mean payoff to group members increases with the proportion of individuals that play dove. Whatever the game, we might expect some subset of individuals to take beneficial actions, and might naively expect a distribution of behaviours such that no individual can do better by changing their action. We refer to such a distribution of behaviours as an equilibrium configuration. In this paper we consider equilibrium configurations when each group member can take one of two actions, where one is beneficial. Under suitable assumptions, we show that in a group at an equilibrium configuration those individuals taking the beneficial action do worse than those taking the other, more selfish, action. For example, in the Producer-Scrounger scenario, scroungers do better than producers. In the Hawk-Dove scenario, hawks do better than doves. Given that these more selfish actions do better, we will refer to them as preferred actions: in a group at the equilibrium configuration each individual should prefer to be one of those taking this action rather than the beneficial action.

In contrast to most models in game theory, the real world is complex [3, 4]. Individuals differ in propensities and experience, so that the social environment is highly variable [5, 6]. Furthermore, environmental conditions fluctuate across generations and vary with spatial location within a generation. Under such circumstance we might expect organisms to acquire information on local social and environmental conditions during development and to modify their behaviour accordingly. That is, we expect adaptive learning.

We will assume that there is no individual recognition in our group setting. Nevertheless, group members can potentially learn about the reward structure in their competitive interactions. Here we assume that each population member continually updates its estimates of the mean reward from each of the two available actions. We focus on a simple reinforcement learning rule that bases the current choice of action on the difference in the current estimates; choosing the one with the higher estimate most of the time, but with occasional choice of the other action in order to sample.

We might expect natural selection to favour mechanisms of learning that result in an accurate representation of the environment. For the simple rule that we consider, this would lead to each population member choosing the action that gives the highest fitness payoff given the behaviour of other population members. In other words, we would expect learning by group members to lead to an equilibrium configuration. As we illustrate, this means that some individuals take the beneficial action most of the time, and hence have a lower payoff than those taking the preferred action. A consequence is that this simple learning rule is not evolutionarily stable: it can be invaded by other learning rules which bias behaviour towards the preferred action. Various forms of bias can be envisaged, but we would expect all to bias behaviour in the same direction. Here we explore a particular form of bias in which the rewards assigned are not the true fitness payoffs but are distorted by an inflation bias. We consider the evolution of this inflation bias, showing that the bias can be substantial for small groups. In other words, we show that it can be rational, in the sense of maximising fitness [7], to have biases when there is learning in a social context during development: learning involving a cognitive bias can be given an adaptive explanation (cf. [8–10]).

Our work is related to that on the evolution of preferences in the economics literature [11]. This indirect evolutionary approach, which can be traced to Guth [12–14], makes a distinction between actual payoffs in terms of fitness and how agents perceive them. Economists refer to such perceptions as preferences; we call them perceived rewards. The approach shows that evolution can favour perceived rewards that do not match payoffs [11, 15, 16], i.e. biased perceptions can evolve. The relationship between biases and rationality is succinctly expressed as ‘Nature can thus mislead her agents, in that preferences and fitnesses can diverge, but cannot mislead herself, in that high fitness wins the day’ [17]. We present examples that have this relationship. Our contribution is to expose the logic of the asymmetries that exist in many common biological games by introducing the idea of equilibrium configurations and preferred actions. We then highlight the role of reinforcement learning in producing best responses and hence establishing such configurations, leading to the need to bias rewards during learning. Furthermore, we show the strong effects of group size on biases that evolve.

## Three focal games

We consider three forms of competition, where in each scenario an individual has a choice between one of two options.

### Hawk-Dove game [18]

Two individuals compete for a resource item of fitness value *V*. In each contest each animal either plays hawk or dove. If both play hawk the contestants fight; the winner takes the item and the loser pays fitness cost *C*. If one plays hawk and the other plays dove the hawk gains the item. If both play dove one of the contestants, chosen at random, gains the item.

### Resource exploitation game

Each member of a group of *G* individuals can either gain a resource of unit value by staying at home and foraging alone or go to a common resource and forage socially. At the common resource all individuals that visit have an equal share of the *V* resources present.

### Producer-Scrounger game [1, 2].

Each member of a group of *G* individuals can either pro-duce or scrounge, although there is at least one producer. Each producer searches for food, finding food sources as a Poisson process with unit rate. Once a producer finds a food source it eats an amount *a* before all the scroungers arrive. It then shares the remaining amount *A* with the scroungers.

## Preferred actions

In many games, including the three focal game scenarios, there is an asymmetry in the two actions when the group is small that arises because the number of organisms in a group is an integer. In order to explore this asymmetry, denote the two possible actions in a round of the game by *u*_1_ and *u*_2_. The fitness payoff to an individual depends on its action and the numbers of other group members playing each of the two actions: specifically if an individual takes action *u*_*i*_ then its payoff is *W*_*i*_(*k*) when *k* other group members take action *u*_2_ and the remaining *G* − 1 − *k* take action *u*_1_. We make two assumptions about the structure of the game.

**A1. Negative frequency dependence.** We assume that the advantage of action *u*_1_ over *u*_2_ increases as the number of individuals taking action *u*_2_ increases. Formally:

A1(i) *W*_1_(*k*) − *W*_2_(*k*) is a strictly increasing function of *k*.
A1(ii) *W*_1_(0) < *W*_2_(0) and *W*_1_(*G* − 1)>*W*_2_(*G* − 1), so that action *u*_2_ is best when none of the other group members take this action and action *u*_1_ is best when all other group members take action *u*_2_.

**A2. Action *u*_2_ is beneficial.** We assume that the payoff to an individual choosing action *u*_2_ never decreases with the number of other group members taking this action; i.e. *W*_2_(*k*)≥*W*_2_(*k* − 1) for all *k* ≥ 1. Note that by the first assumption this means that *W*_1_(*k*) is a strictly increasing function of *k*.

These assumptions hold for the Hawk-Dove game where *u*_1_ = hawk, for the Resource Exploitation game where *u*_1_ = forage socially, and, providing the group size *G* is sufficiently large, holds for the Producer-Scrounger game where *u*_1_ = scrounge (Appendix A in S1 Appendix).

The following result can be established for the generic case (Appendix A in S1 Appendix).

1. **Equilibrium configuration.** There exists a unique number k* such that no individual can do better by changing its action when *k*8 individuals take action *u*_2_ and *G* − *k*8 take action *u*_1_.
2. **Preferred action.** At the equilibrium configuration, individuals taking action *u*_1_ receive the higher payoff.

**Fig 1:**
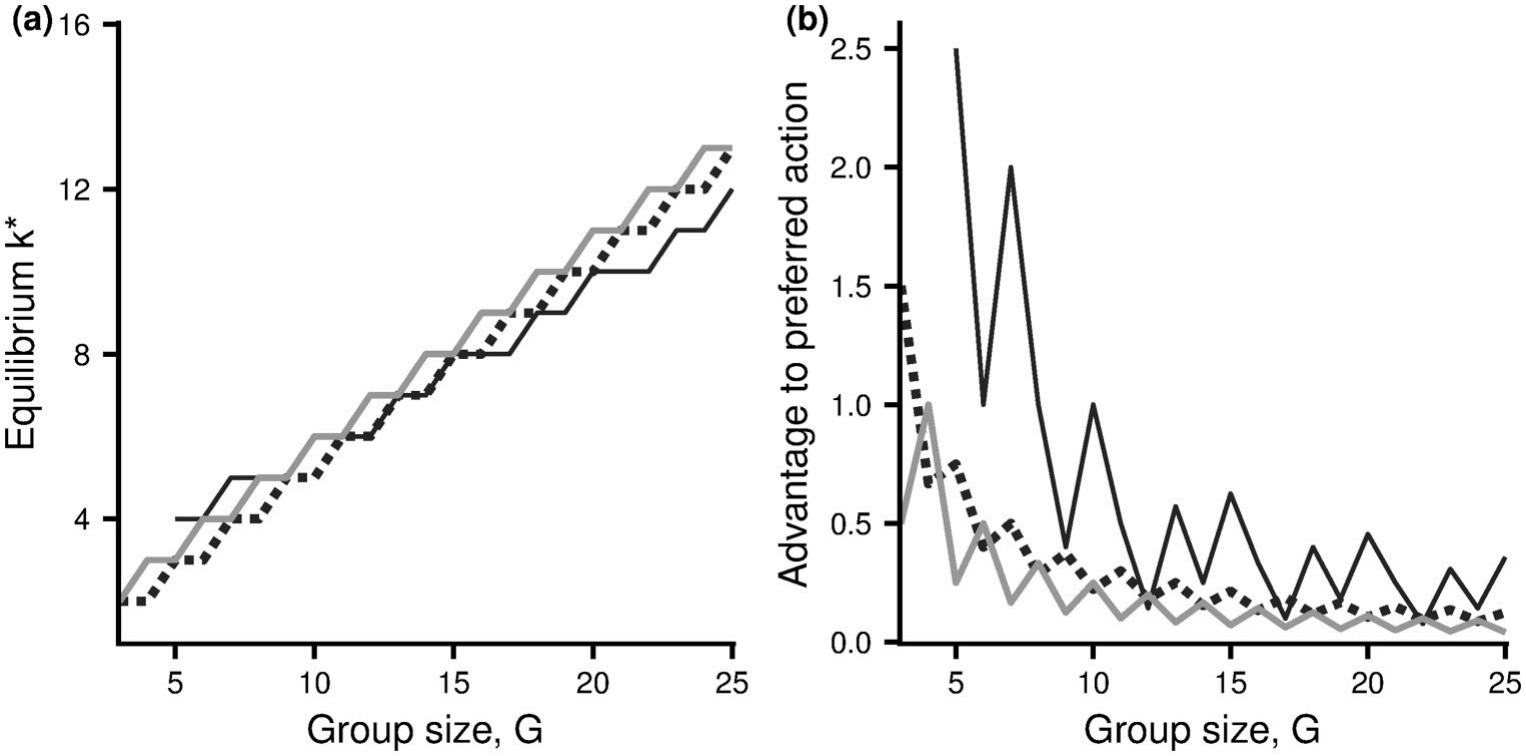
Equilibrium configurations and the advantage of preferred actions as a function of group size. (a) The number of individuals, k*, taking the beneficial action at the equilibrium configuration. (b) The payoff advantage under the preferred action at this equilibrium. Dashed curve: Hawk-Dove game with *V* = 2, *C* = 4. Light grey solid curve: Resource Exploitation game with *V* = 0.5*G*. Dark grey solid curve: Producer-Scrounger game with *a* = 2, *A* = 3. For this latter game there is no equilibrium configuration for *G* ≤ 4 (Appendix B in S1 Appendix).

In the light of this result we will refer to action *u*_1_ as the preferred action and *u*_2_ as the beneficial action. Thus hawk is the preferred action in the Hawk-Dove game and social foraging is the preferred action in the Resource Exploitation game. In the Producer-Scrounger game, an equilibrium configuration only exist for sufficiently large group size. When it does so, scrounge is the preferred action. The equilibrium configuration and the advantage of taking the preferred action at this configuration are illustrated in Fig 1 for each of the three scenarios.

## Learning the best action

We investigate how an animal might learn which is the best action to take given the behaviour of other group members when each individual plays many rounds of a game during an extended period. A population of size *N* = 15000 is subdivided into *N*/*G* local groups of size *G* (*G* ≥ 3). Members of each group play *K* rounds of contests against one another (Appendix B in S1 Appendix). There is no individual recognition of other group members, and so no reputation effects.

We consider a simple learning rule. For the Hawk-Dove and Resource Exploitation scenarios the rule is as follows. Let *n*_*i*_(*t*) denote the number of times action *u*_*i*_ is taken during the first *t* rounds. Let *r*_*i*_(*t*) denote the total reward obtained in these *n*_*i*_(*t*) rounds. Set

$$\begin{array}{c} R_{i}\left(t\right)=\frac{r_{0}+r_{i}\left(t\right)}{1+n_{i}\left(t\right)}, \end{array}$$ (1)

where *r*_0_ is a constant. An individual takes action *u*_2_ in round *t* + 1 with a probability *f*(*R*_2_(*t*) − *R*_1_(*t*)) that is a function of the difference in these ‘rates’. Here the function *f* is an increasing function that satisfies *f*(1 − *x*) = 1 − *f*(*x*) and lim_*x* → ∞_ *f*(*x*) = 1 (Appendix B in S1 Appendix). This function is close to a step function, so that most of the time an animal chooses the action with the greatest value of *R*, although they occasionally sample by taking the action with the lower value of *R*. The constant *r*_0_ is the same for each action and is set to be positive (Appendix B in S1 Appendix). Its effect is to promote initial sampling.

For the Producer-Scrounger scenario the leaning rule is the analogous rule but with the time spent on each action replacing the number of times the action has been taken (Appendix B in S1 Appendix).

**Fig 2:**
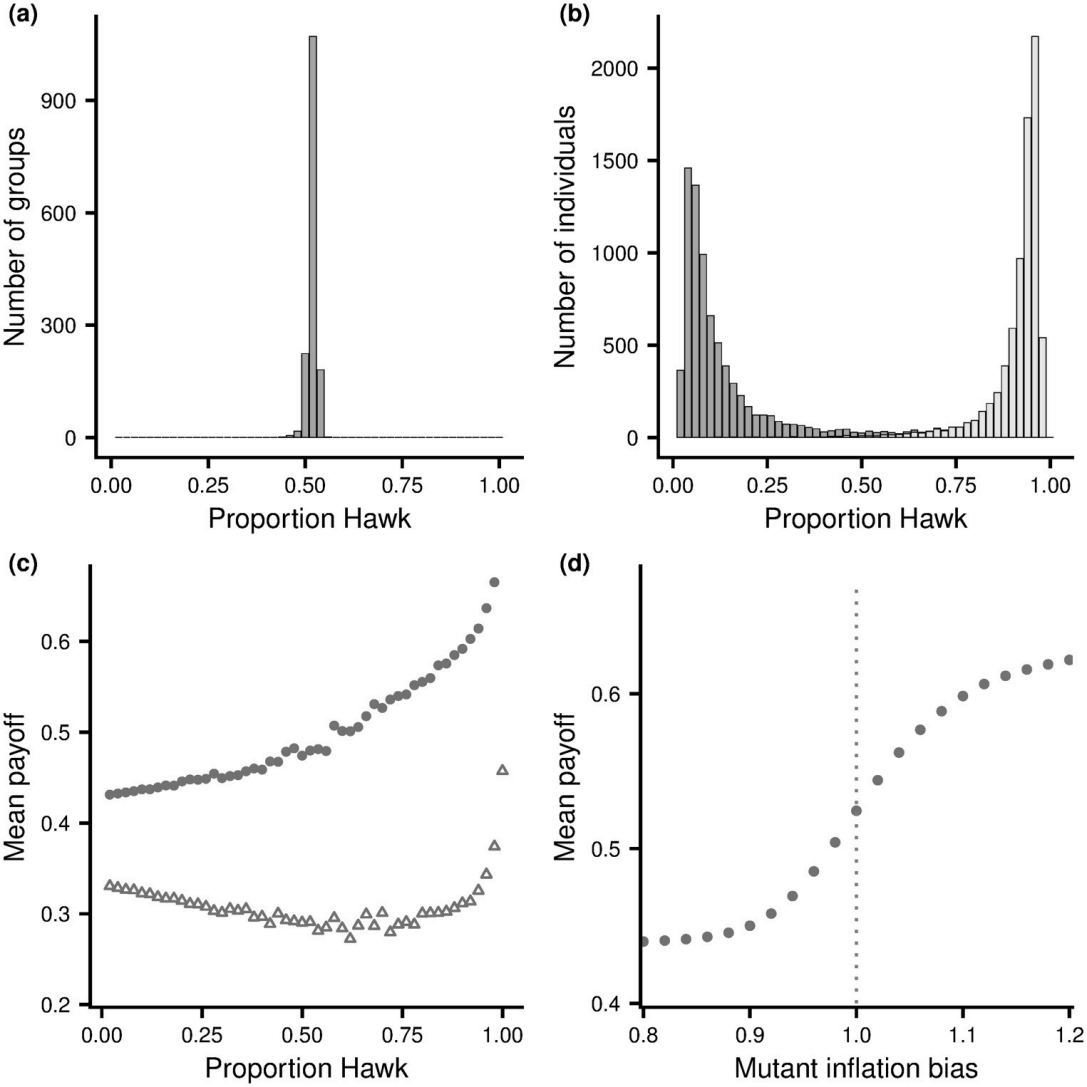
Learning outcomes for the Hawk-Dove game. A group of 15000 individuals is divided into 1500 groups of size *G* = 10. Members of each group play repeated rounds of the Hawk-Dove game against one another, using the simple learning rule (Appendix B in S1 Appendix). In (a) and (b) learning is based on rewards that are fitness payoffs. (a) The number of groups where the overall proportion of choices that are hawk made by members of the group take a given value. (b) The number of individuals that play hawk a given proportion of the time, subdivided into those that have a greater fitness payoff per round when playing dove (dark grey) and those that have greater payoff per round when playing hawk (light grey) (c) The mean fitness payoff of individuals that play hawk a given proportion of the time: with learning based on unbiased rewards; i.e. fitness payoffs (filled circles), and after evolution of inflation bias (open triangles). (d) The mean fitness payoff to a mutant with given inflation bias *α* when all other population members use unbiased rewards (*α* = 1), with each point estimated from 100000 independent simulations of bouts of group learning. Parameter values *V* = 2, *C* = 4.

Fig 2(a) illustrates the results of employing this learning rule for the Hawk-Dove game, when rewards are the fitness payoffs. For the case illustrated (groups of size *G* = 10), at the equilibrium configuration k* = 5 members of a group play hawk (Fig 1(a)). For almost all groups, the proportion of all choices by group members that were hawk is very close to 0.5 (Fig 2(a). Furthermore, most population members spend most of their time taking the same action, with close to one half choosing dove most of the time and half choosing hawk (Fig 2(b). These results are consistent with learning leading to an equilibrium configuration within each group. Fig 2(b) shows that those individuals that predominantly choose dove do better when they choose dove and those that predominantly choose hawk do better when they choose hawk. This reinforces the idea of an equilibrium configuration and suggests that the learning rule is working as intended: individuals are learning to take the best action given the actions chosen by others. Fig 2(c) shows that the mean payoff to a population member increases with the frequency with which it chooses hawk. Thus, although individuals are learning to play the best action given the behaviour of others in the group, those that learn to play the preferred action (hawk) are doing better.

Analogous results hold for the Resource Exploitation and Producer-Scrounger scenarios (S1(a)–S1(c) Fig in S1 Appendix and S2(a)–S2(c) Fig in S1 Appendix). In particular at the equilibrium configuration, social foragers do better in the Resource Exploitation scenario and scroungers do better in the Producer-Scrounger scenario.

**Fig 3:**
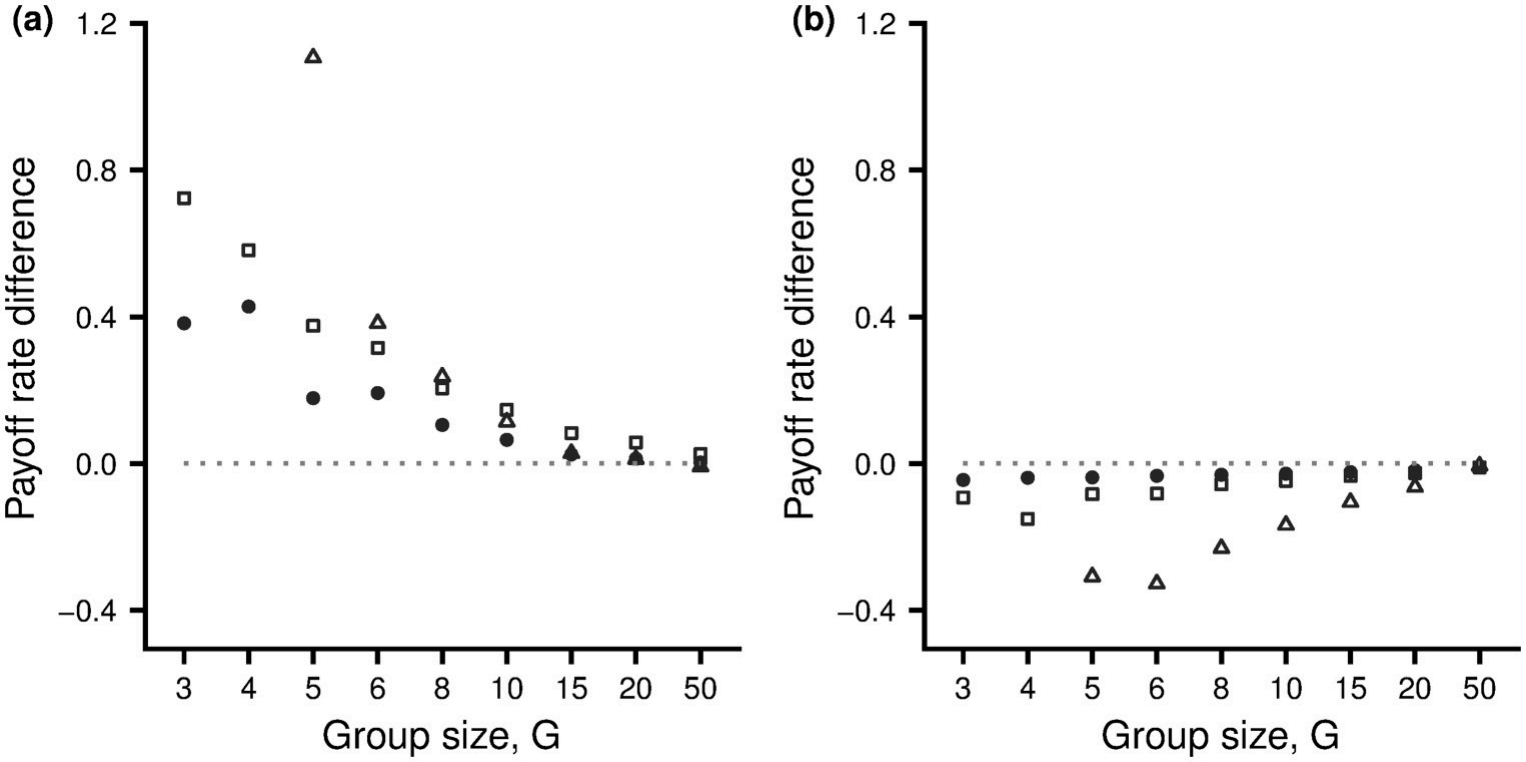
Differences in payoff rates. The population average difference in rate of fitness payoff between the preferred action and the beneficial action, as a function of group size *G*. (a) Subjective rewards are unbiased (*α* = 1). (b) After the inflation factor *α* has evolved for 10000 generations. The three cases shown are: Hawk-Dove game (open squares), Resource Exploitation game (filled circles) and Producer-Scrounger game (open triangles). For the latter game, the cases *G* = 3, 4 are not shown as no equilibrium configuration exists. For each value of *G*, a group of 15000 individuals is divided into $\frac{15000}{G}$ groups of size *G*. Game parameter values as for Fig 1.

The advantage of playing the preferred action is also illustrated in Fig 3(a), which shows the average difference in payoff rates under the two actions. In all cases where an equilibrium configuration exists, the payoff rate under the preferred action is greater than under the beneficial action. Differences diminish as group size increases.

## Biased subjective rewards

The above suggests that if group members repeatedly play one of the focal games with other group members, then each should try to induce others to take the beneficial action *u*_2_ so that it can take the preferred action *u*_1_. Here we investigate one mechanism, based on learning with biased subjective rewards, that can achieve this goal.

We allow the subjective rewards in a round to be different to the fitness payoffs by introducing an inflation bias *α* (where *α* > 0) as follows (see also Appendix B in S1 Appendix). In the Hawk-Dove scenario a resource of fitness value *V* has subjective reward *αV*, whereas losing a fight has subjective reward equal to its true value −*C*. Thus when *α* > 1 resources are more subjectively rewarding than their true value, or equivalently the cost of losing a fight is subjectively devalued. Conversely, if *α* < 1 the cost of losing a fight is subjectively overvalued. In the Resource Exploitation game, if actual resource *v* is obtained from the social resource the subjective reward is *αv*. In contrast, the subjective reward from food found on the home territory is its true resource value of 1. In the Producer-Scrounger game food found as a scrounger is subjectively inflated by the factor *α*, while food found as a producer is not inflated. In all three scenarios, the preferred action is overvalued when *α* > 1.

During learning, the reward rates *R*_1_ and *R*_2_ are now formed using the subjective rewards rather than payoffs. When *α* ≠ 1 we will refer to subjective rewards as biased. We investigate the advantage of biasing by assuming that a single individual has biased subjective when the rest of the population have unbiased subjective rewards (*α* = 1). Fig 2(d) illustrates the effect of the inflation bias *α* in the Hawk-Dove scenario. As can be seen, if the individual overvalues the reward from a contest relative to the cost (*α* > 1), it has a greater payoff rate than the rest of the population. Increasing the inflation bias results in the individual playing hawk more often. Other group members then have a reduced payoff if they play hawk, and so learn to play hawk less often, to the benefit of the focal individual.

The focal individual is effectively playing against the entire rest of the group here, but there is direct analogy with equilibrium behaviour in a two-player game. If the two-player game is played with simultaneous choice, then at the Nash equilibrium each player is taking the best action given the action of the opponent. In the Stackelberg version of the game Player 1 chooses first, with Player 2 making their choice in response to the action of Player 1. In this latter version of the game it is optimal for Player 2 to take the best action given the action of Player 1. In many games, this can be exploited by Player 1, so that this player does better than at the Nash equilibrium for the simultaneous version of the game [19]. At the Stackelberg equilibrium, Player 1 is taking the best action given that Player 2 will adjust their action, but at this equilibrium the action of Player 1 is typically not the best if the action of Player 2 were held fixed. In our group setting, other group members, who are using unbiased subjective rewards, learn to take the best action given that of other group members, and are analogous to Player 2. The focal individual is analogous to Player 1. This individual exploits the best-response behaviour of other group members to improve its own payoff. However, in order to do so it often chooses to play hawk when this will yield a lower mean immediate payoff than playing dove, but this is more than made up for by the adjustment in future behaviour by other group members.

Similar effects of inflation bias occur in the Resource Exploitation scenario (S1(d) Fig) in S1 Appendix and in the Producer-Scrounger scenario (S2(d) Fig in S1 Appendix). In the Resource Exploitation case a bias towards social foraging reduces the payoff to others if they also forage socially, so deterring others, to the advantage of the focal individual. In the Producer-Scrounger case a bias towards scrounging leads to more of the others producing, again to the advantage of the focal individual.

## The evolution of bias

In order to investigate the evolution of the inflation bias *α* we assume that a population of fixed size *N* has non-overlapping generations. In each generation the population is subdivided into *N*/*G* local groups of size *G* with group members playing *K* rounds of a contest against one another using the above learning rule. The fitness of an individual is proportional to the individual’s rate of payoff (i.e. its true net rate of resource gain), plus a background term. We treat *α* as a quantitative trait and assume that there is sexual reproduction (but only one mating type). In this reproductive phase each new member of the subsequent generation has two parents with each parent chosen independently from the population with a probability proportion to its fitness. Inheritance is described by the infinitesimal model [20], so that the inflation bias of the offspring is equal to the mean bias of the two parents plus an error with zero mean and standard deviation *σ* = 0.02.

**Fig 4:**
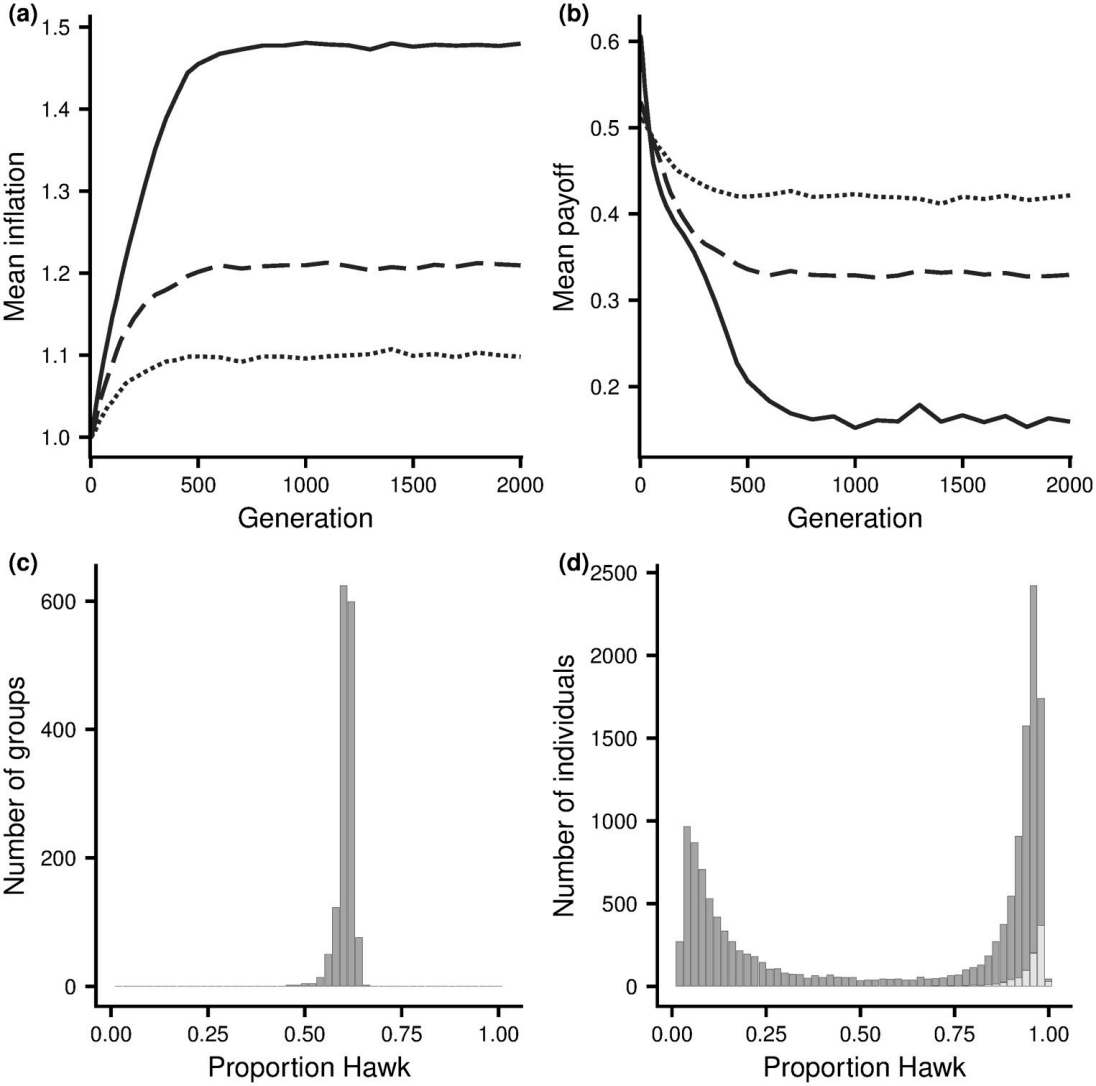
Evolution of the inflation bias for the Hawk-Dove game. (a) Evolved mean bias. (b) The mean fitness payoff of population members. (c) and (d) are the analogues, after evolution of inflation bias, of Fig 2 panels (a) and (b), respectively. In (a) and (b), group sizes are: *G* = 5 (solid curves), *G* = 10 (dashed curve) and *G* = 20 (dotted curve). In (c) and (d), *G* = 10. Parameters *V* = 2, *C* = 4.

**Fig 5:**
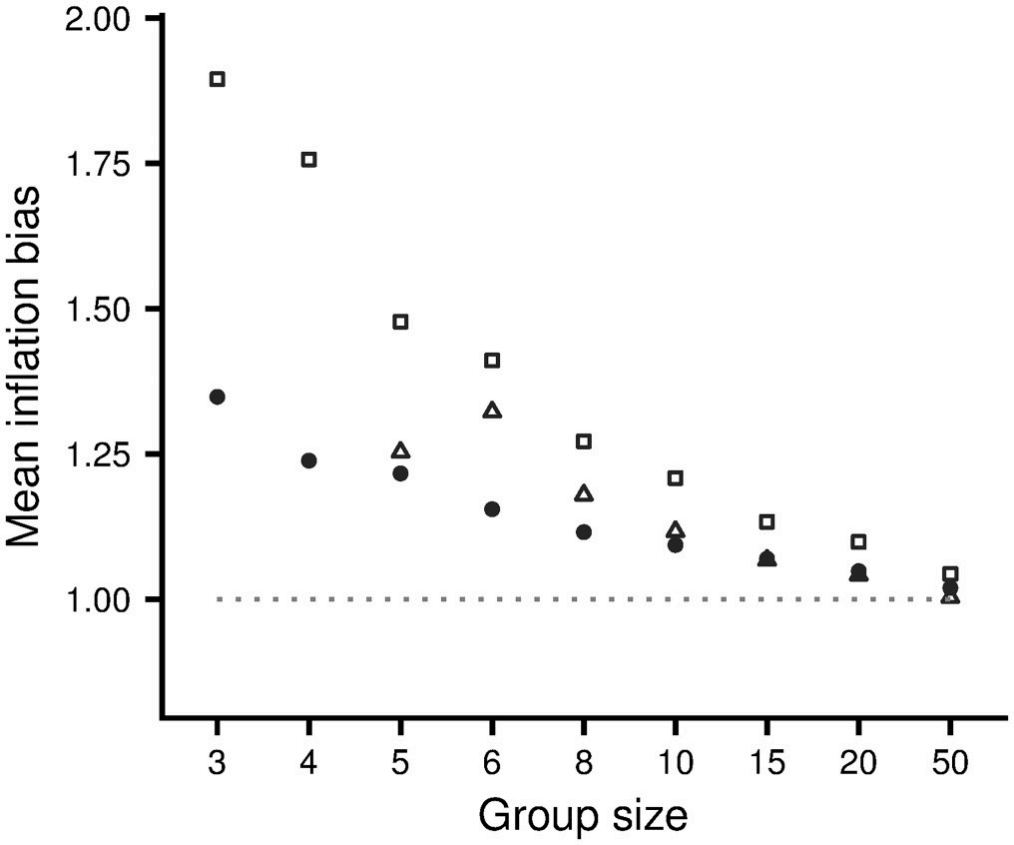
Evolved inflation biases for the three scenarios. Open squares: the Hawk-Dove game with *V* = 2, *C* = 4. Solid circles: the Resource Exploitation game with *V* = 0.5*G*. Open triangles: the Producer-Scrounger game with *a* = 2, *A* = 3. In this latter game there is no equilibrium configuration for *G* ≤ 4, and the mean inflation bias evolves to less than 0.75 (not shown). Results are averages over generations 8000 to 10000.

Fig 4(a) illustrates the evolution of inflation bias for the Hawk-Dove game. The corresponding results for the other two scenarios are illustrated by S3 and S4 Figs in S1 Appendix. In all scenarios there is rapid evolution to stable levels of inflation bias, although there are always fluctuations about the mean value over time and genetic variation about the mean value at a given time (within-generation standard deviations are not shown but always lie between 0.03 and 0.04). Fig 5 shows the dependence of evolved levels of inflation bias on group size, for the three scenarios. For small groups, evolved inflation biases are significantly greater than 1. Biases decrease as group size increases. When groups are large (*G* = 50) biases are close to 1, as we would expect, giving confidence that the effect in smaller groups is not just an artefact of the learning rule employed.

For the Hawk-Dove scenario, in the evolved population individuals overvalue the resource relative to the cost of a fight. Learning results in members of a group having an approximate equilibrium configuration for this biased reward structure. As a consequence, evolution of inflation bias leads to a greater tendency to play hawk (compare Fig 2(a) with Fig 4(c) and Fig 2(b) with Fig 4(d)). At this new equilibrium configuration the (true) advantage of those that play hawk over those that play dove has disappeared (Fig 2(c)). Although individuals in a group are doing the best given their biased rewards, they are no longer doing the best given the true reward structure, with most experiencing a slightly higher payoff rate from playing dove than playing hawk (Fig 3(b)). Despite the higher payoff rate from playing dove, it would not benefit an individual to increase the frequency with which it takes this action as other group members would then learn to play Hawk more often. This is in line with our previous remarks on Stackelberg equilibria. Exactly analogous results hold for the other two game scenarios (see S1 Appendix).

Since evolution results in a reduced propensity to take the beneficial action, mean population fitness decreases in the Hawk-Dove scenario (Fig 4(b)) and in the other two scenarios (S3(b) and S4(b) Figs in S1 Appendix).

## Discussion

We have been concerned with a group of individuals playing a game with two actions. At an equilibrium configuration for the group, no individual can improve its fitness payoff by unilaterally changing its action. Under simple assumptions on negative frequency dependence and the prosocial effects of a beneficial action, we have shown that at an equilibrium configuration those individuals taking the other more selfish action (which we refer to as the preferred action) have a greater payoff. We then considered how individuals in a group might learn which action to take if there are many rounds of the game. Learning based on true fitness payoffs results in an equilibrium configuration, and so leads to those that end up taking the preferred action doing better. This selects for some form of bias towards taking the preferred action. We have considered a specific form of bias, showing that at evolutionary stability there can be a considerable difference between true fitness payoffs and evolved subjective rewards.

Our general conclusion is that the difference between the two actions is reflected by an asymmetry in how rewards from the two actions are valued; in other words we show that a biased evaluation is rational. There is considerable interest in the evolution of biases [8–10, 21], including investigations of overconfidence and optimism. [22] note that overconfidence and optimism are often used in vague or misleading ways. In contrast, [23] define optimism and pessimism in terms of the weight that an agent gives to the consequences of its behaviour for any choice by the other player. An optimist overestimates this and a pessimist underestimates it. Optimism is the general evolutionary outcome [23].

Johnson and Fowler [24] show that overconfidence can evolve when two individuals contest a resource in a variant of the Hawk-Dove game. [25] argue that this result relies on constraining an individual to use just its probability of winning a fight to decide whether to contest a resource. Given this constraint, the appropriate behaviour can only be achieved by distorting the estimated probability of winning. Without the constraint, decisions can be explicitly based on *V* and *C*, as they are in our current analysis of the Hawk-Dove game. Our approach follows the standard indirect evolutionary approach of basing decisions on perceived rewards and allowing these to evolve [11, 15, 16, 23]. Because we find that biases emerge in this general context, we regard them as genuine whereas the overconfidence found by [24] is an artefact of the constraint. Although *V* and *C* influence behaviour [26] this does not undermine the argument that the only evolutionary option in the model of Johnson and Fowler is to modify the estimated probability of winning [25]. Johnson and Fowler support their model by arguing that it is based on a simple heuristic that can generate a rapid decision. Although we agree that plausible decision rules need to be relatively simple [3, 27, 28], it is important to maintain a distinction between biases that are rational in the sense that they emerge in a general framework and effects that result from heuristics that will not always achieve an evolutionarily stable outcome [9, 25].

It has been suggested that learning in games might be based on the same general adjustment mechanisms that are used in situations that do not involve interactions with other agents (i.e. games against nature) [29, 30] and the performance of various simple rules has been investigated in both cases [29, 31, 32]. In contrast to this view, learning during development often takes place in situations in which there is a small and fairly stable group of other individuals that are also learning as they develop. In such a group, individuals that use unbiased subjective rewards will tend to do the best given the behaviour of others, so that an equilibrium configuration will result. At this configuration some individuals will do worse than others, leading to selection on biasing subjective rewards in favour of taking the preferred action. Put another way, a rule which learns to do the best given the behaviour of others can be exploited (e.g. [33–35]). This suggests that the parsimonious assumption that the same rule can be applied to both games against nature and games against other agents might not be valid. The problem is that rules that learn about the consequences of actions and use this information to maximise payoff are fine in a non-game theoretical context, but in a game current behaviour has two consequences (i) it yields immediate payoffs, and (ii) it affects future payoffs because it affects the experience of other group members and hence, since they learn, affects their future behaviour [16, 35, 36]. It may then be worth taking an action that does not maximise the current payoff if the loss in current payoff is more than made up for in the future. On this argument we expect a dilution of the effect on others as group size increases, and this is what we find.

We have analysed three scenarios in which inflation bias of payoffs acts as a mechanism to prevent exploitation, and to achieve the correct balance between immediate payoffs and future gains through the effect on others. [33] illustrates a related case of exploitation in a game in which each of two parents chooses the effort expended on care of their common young. Parents respond to the efforts of one another. As [33] shows, if parents always adjust their efforts to be the best given the effort of partner, the population is not evolutionarily stable. Stability is achieved if each prevents exploitation by behaving as if they are less able to care than is the case. Similarly, in the public good game analysed by [34] group members behave as if they are less able to contribute to the common good than is the case.

What matters for evolution is how an individual behaves. As [37] point out, there can be more than one way to achieve the appropriate behaviour. For example, in the context of human dating, a male could overestimate his attractiveness or ask a woman for a date even if he knows that he is not very attractive. In the Hawk-Dove game, the optimal decision depends on *V*, *C* and the probability of winning; each could be modified to obtain a given outcome. Similarly, in our models, different forms of inflation bias could be favoured. In particular, in our three scenarios there was considerable time to learn during a generation. In situations in which less time is available the bias might be on the prior propensity to perform the preferred action. Whatever the form of bias, if agents are learning then some form of bias is necessary to avoid exploitation.

Other assumptions concerning the genetics of the inflation bias trait are also possible. However, given the selection pressure to increase inflation bias shown in Fig 2(d) (S1(d) and S2(d) Figs in S1 Appendix) we would expect inflation bias to initially increase under any reasonable genetic model. Our infinitesimal model precludes a polymorphism in the final evolved population. Other assumptions may lead to other final outcomes, and the effect of the genetic system might be investigated in future work.

We show the “bias” effect for games that have a “preferred action” played in small groups. Is there evidence that the rule used depends on context? [30] argue for the use of the same rule in social games and games against nature. Other evidence suggests that games are special [38] and there might be separate areas of the brain for dealing with rewards and social interactions [39–41]. The brain activity of humans playing the Ultimatum game or the Prisoner’s Dilemma game depends on whether they are told that they are playing against a human opponent rather than a computer [42]. When rhesus monkeys play a matching game against a computer they learn to avoid being exploited. This involves departure from simple learning rules [40]. The level of cooperation shown by chimps in the Stag Hunt game depends on how easy it is for them to see their partner [43]. We conclude that it is plausible that some species treat social games and games against nature in different ways. It remains to be seen if there is an association between particular games and rules.

An agent has a theory of mind (TOM) if it is able to represent the beliefs of other agents [41, 44]. In the context of the repeated Prisoner’s Dilemma game, [45] argues that unless an agent has a TOM it can be exploited by another agent; specifically because a TOM is required in order to anticipating the long-term consequences of current behaviour. In contrast, our results suggest that exploitation can be avoided without the need for a sophisticated TOM by simply affecting the future through the use of bias of current rewards. It is not even necessary for agents to recognise each other.

## Supporting information

S1 Appendix (PDF)
