# Supplementary material for: Learning, exploitation and bias in games
Source: PLoS One. 2021 Feb 5;16(2):e0246588. doi: 10.1371/journal.pone.0246588 (PMC7864454; doi:10.1371/journal.pone.0246588)
Supplement: S1 Appendix — (PDF) [file pone.0246588.s001.pdf]

S1 Appendices

Appendix A. Preferred actions

By assumption the function $D(k) \equiv W_1(k) - W_2(k)$ of k is strictly increasing and satisfies $D(0) < 0$ and $D(G-1) > 0$. We begin by assuming that $D(k) \neq 0$ for any k. We refer to this as the generic case.

In the generic case there is a unique k^* satisfying the two conditions $D(k^*-1) < 0$ and $D(k^*) > 0$. Consider a group in which k^* individuals take action u_2. Then the first of these conditions says that

$$W_2(k^*-1) > W_1(k^*-1), \tag{S1}$$

so that an individual that takes action u_2 get a strictly lower payoff by switching to action u_1. The second condition says that

$$W_2(k^*) < W_1(k^*), \tag{S2}$$

so that an individual taking action u_1 gets a strictly lower payoff by switching to action u_2. It can also be seen that k^* is the unique value of k for which it is detrimental for any group member to switch action.

At the equilibrium configuration, the payoff to an individual choosing action u_1 is $W_1(k^*)$. Similarly, the payoff to individual choosing action u_2 is $W_2(k^*-1)$. We have

$$\begin{aligned} W_1(k^*) &> W_2(k^*) \quad \text{by equation S2} && \text{(S3)} \\ &\geq W_2(k^*-1) \quad \text{by our assumption that action } u_2 \text{ is beneficial} && \text{(S4)} \end{aligned}$$

Thus those taking action u_1 do strictly better than those taking action u_2. We will refer to action u_1 as the preferred action.

In the non-generic case there is an integer $\hat{k}$ such that $D(\hat{k}) = 0$. There are then two possible definitions of k^*, namely $k^* = \hat{k}$ and $k^* = \hat{k} + 1$.

First consider a group in which $k^* = \hat{k}$ individuals take action u_2. Then an individual taking action u_1 gets the same payoff by switching to action u_2, while an individual taking action u_2 gets a strictly lower payoff by switching to u_1. In this group $W_1(k^*) \geq W_2(k^*-1)$, and this inequality is strict if the payoff $W_2(k)$ is strictly increasing with k.

Now consider a group in which $k^* = \hat{k} + 1$ individuals take action u_2. Then an individual taking action u_1 gets a strictly lower payoff by switching to action u_2, while an individual taking action u_2 gets the same payoff by switching to u_1. In this group $W_1(k^*) > W_2(k^*-1)$.

Thus in the non-generic case the equilibrium configuration is not unique, but at either configuration, those taking action u_1 do strictly better than those taking action u_2 provided $W_2(k)$ is strictly increasing with k (as is true in the Hawk-Dove game and the Producer-Scrounger game). The analyses below and computations assume the value $k^* = \hat{k} + 1$ when payoffs are non-generic.

The Hawk-Dove game. We consider the standard Hawk-Dove game with value of reward, V, and cost of losing a fight, C. We assume that $V < C$. The actions are $u_1 =$ Hawk, and $u_2 =$ Dove. Payoffs are

$$W_1(k) = \frac{k}{G-1}V + \left(1 - \frac{k}{G-1}\right)(V-C)/2 = \frac{1}{2}\left[(V-C) + \frac{k}{G-1}(V+C)\right], \tag{S5}$$

$$W_2(k) = \frac{1}{2}\frac{k}{G-1}V. \tag{S6}$$

From these payoffs we have

$$2(W_1(k) - W_2(k)) = (V - C) + \frac{k}{G-1}C. \tag{S7}$$

Thus conditions A1 and A2 of the main text are satisfied since $W_1(k) - W_2(k)$ and $W_2(k)$ are strictly increasing functions of k.

Let k^* be the minimum value of k such that $W_1(k) > W_2(k)$, so that

$$k^* = 1 + \text{ integer part of } \quad \left[(G-1)(1-\frac{V}{C})\right]. \tag{S8}$$

The advantage of a Hawk over a Dove at the equilibrium configuration is

$$\kappa(G) = W_1(k^*) - W_2(k^* - 1) \tag{S9}$$

$$= \frac{1}{2(G-1)}\left[GV - (G-1-k^*)C\right]. \tag{S10}$$

The resource exploitation game. Each group member can either obtain a resource in a communal place (the social foraging action u_1) or its own territory (the solitary foraging action u_2). All those choosing the communal place share a resource of value V equally. An individual that chooses it own territory gains unit resource. Payoffs are

$$W_1(k) = \frac{V}{G-k}, \tag{S11}$$

$$W_2(k) = 1. \tag{S12}$$

We assume that $1 < V < G$. Under this assumption, assumptions A1 and A2 both hold.

We have

$$k^* = 1 + \text{ integer part of } (G - V). \tag{S13}$$

The advantage of a social over a solitary forager at the equilibrium configuration is

$$\kappa(G) = W_1(k^*) - W_2(k^* - 1) \tag{S14}$$

$$= \frac{V}{G-k^*} - 1. \tag{S15}$$

The Producer-Scrounger game. In this game u_1 = Scrounge, and u_2 = Produce. Payoffs are

$$W_1(k) = \frac{Ak}{G+1-k}, \tag{S16}$$

$$W_2(k) = a + \frac{A}{G-k}. \tag{S17}$$

As can be seen, both of these payoffs are strictly increasing functions of k. Thus producing is beneficial.

In order to check the negative frequency dependence assumption we set $D(k) = \frac{1}{A}(W_1(k) - W_2(k))$, so that

$$D(k) = \frac{k}{G+1-k} - \frac{1}{G-k} - \frac{a}{A}. \tag{S18}$$

Then for $0 \leq k \leq G-2$ we have

$$D(k+1) - D(k) = \frac{k+1}{G-k} - \frac{1}{G-1-k} - \frac{k}{G+1-k} + \frac{1}{G-k} \tag{S19}$$

$$= k\left[\frac{1}{G-k} - \frac{1}{G+1-k}\right] \tag{S20}$$

$$+ \frac{1}{(G-k)(G-1-k)}\left[(G-2)-k\right]. \tag{S21}$$

The term in the first square bracket is non-negative and is positive for $k \geq 1$. The term in the second square bracket is non-negative and is positive for $k = 0$. It follows that $D(k)$ is a strictly increasing function of k.

To check the end conditions we first note that $W_1(0) < W_2(0)$. We also have $W_1(G-1) - W_2(G-1) = \lambda\left(\frac{(G-1)A}{2} - a - A\right)$. Thus to ensure that $W_1(G-1) > W_2(G-1)$ we require that

$$G > 3 + \frac{2a}{A}. \tag{S22}$$

Computations are based on assuming that $a = 2$ and $A = 3$. For these values we require $G \geq 5$.

The condition that $D(k) > 0$ translates into $J_2k^2 - J_1k + J_0 < 0$, where

$$J_0 = (G+1)\left(1 + \frac{a}{A}G\right) \tag{S23}$$

$$J_1 = G + 1 + \frac{a}{A}(2G+1) \tag{S24}$$

$$J_2 = 1 + \frac{a}{A}. \tag{S25}$$

Let the real-valued function d be given by $d(x) = J_2x^2 - J_1x + J_0$. This quadratic function of x satisfies $d(0) > 0$. It also satisfies $d(G-1) < 0$ by condition S22. Let x^* be the smaller of the two reals roots of the equation $d(x^*) = 0$. Then $0 < x^* < G-1$, and we have $d(x) > 0$ for $0 < x < x^*$ and $d(x) < 0$ for $x^* < x \leq G-1$. It follows that

$$k^* = 1 + \text{ integer part of } x^*. \tag{S26}$$

The usual quadratic formula gives

$$x^* = \frac{J_1 - \sqrt{J_1^2 - 4J_2J_0}}{2J_2}. \tag{S27}$$

The advantage of a Scrounger over a Producer at the equilibrium configuration is

$$\kappa(G) = W_1(k^*) - W_2(k^*-1) \tag{S28}$$

$$= \frac{A(k^*-1)}{(G+1-k^*)} - a. \tag{S29}$$

Appendix B. Learning

Time structure

The Hawk-Dove game. Group members are assigned a random ordering, $1, 2, ..., G$. In a cycle of rounds, group member 1 chooses a randomly selected opponent from the other $G - 1$ group members and plays the Hawk-Dove game against this opponent, then group member 2 does the same, and so on until all group members have done so. Thus in a cycle there are $2G$ rounds of the game. Updating of the subjective reward rates occurs after each round. Computations used to produce the figures are based on learning over $K = 10000$ cycles.

The resource exploitation game. During a round of the game each group member decided whether to forage socially or solitarily. The subjective reward rates are updated after each round in the cycle. Computations used to produce the figures are based on learning over $K = 10000$ rounds.

The Producer-Scrounger game. At the start of a round, each group member decides whether to be a producer or a scrounger. If no individual chooses to produce, there is another round of choice. This continues until at least one of the group members chooses to be a producer. This choice phase is instantaneous. Once at least one individual is a producer, all producers search for a food source. We assume that each producer finds food sources as a Poisson process of unit rate, independently of others. Once the first producer finds a food source, this source is consumed by that producer and all the scroungers. This consumption phase is instantaneous. The round then ends and another begins, with all again choosing to be producers or scroungers. Thus exactly one food source is consumed during a round, and the time taken to complete a round has an exponential distribution with parameter equal to the number of producers in that round.

The number of producers tends to increase with group size. Thus the mean time taken for a round tends to decrease with group size. The probability a given producer find a food source in a round also tends to decrease with group size. Because of these effects, we have chosen the duration of the learning phase to decrease with group size. Specifically, in the computations used to produce the figures we have assumed that rounds continue until the total time exceeds $T_{max} = 250 + \frac{15000}{G}$. This is a compromise between having too short a time to learn and have more rounds to learn as group size increases.

Subjective rewards

The Hawk-Dove game. The true reward (payoff) of obtaining the resource in a contest is V. The true reward of losing a hawk vs hawk fight is $-C$. The true reward from failing to obtaining a reward when choosing dove is zero. If the individual has inflation bias α then the subjective rewards for these three outcomes are αV, $-C$ and 0 respectively. Computations used to produce the figures are based on the values $V = 2, C = 4$.

The Resource Exploitation game. The true reward from solitary foraging is 1. When n group members choose to forage socially the true reward to each is $\frac{V}{n}$. If an individual has inflation factor α then the subjective rewards for these two outcomes are 1 and $\alpha\frac{V}{n}$ respectively. Computations used to produce the figures assume that $V = 0.5G$.

The Producer-Scrounger game. In a round of this game any producer that does not find a food source has true reward zero. The producer that finds the food source has true reward $a + \frac{A}{n}$ when there are n scroungers in the group. The true reward to each scrounger is $\frac{A}{n}$. The subjective reward to a producer is the true reward. The subjective reward to a scrounger is α times the true reward. Computations used to produce the figures assume that $a = 2, A = 3$.

The choice rule

Let $w_i(s)$ to be the subjective reward on round s if action u_i is chosen on this round, with $w_i(s) = 0$ if the other action is chosen. Let

$$r_i(t) = \sum_{s=1}^{t} w_i(s) \tag{S30}$$

be the total subjective reward from action u_i in the first t rounds. Consider first the Hawk-Dove and Resource Exploitation scenarios. Let $n_i(t)$ be the number of times action u_i is chosen in the first t rounds. Then after t round the subjective rate of reward under action u_i is set to be

$$R_i(t) = \frac{r_0 + r_i(t)}{1 + n_i(t)}. \tag{S31}$$

Computations used to produce the figures assume that $r_0 = 50$.

For the Producer-Scrounger scenario, let $\tau_i(t)$ denote that total time devoted to action u_i in the first t rounds. Then after t round the subjective rate of reward under action u_i is

$$R_i(t) = \frac{r_0 + r_i(t)}{1 + \tau_i(t)}. \tag{S32}$$

Computations used to produce the figures assume that $r_0 = \frac{T_{max}}{20}$.

The probability that the individual chooses action u_2 on round $t+1$ is a function, $f(R_2(t) - R_1(t))$, of the difference between the current subjective rates. Computations are based on the function $f(d) = 0.5[(J + 2|d|)/(J + |d|)]$ for $d \geq 0$ and $f(d) = 1 - f(-d)$ for $d < 0$. We have taken $J = 0.005$ in computing figures for the Hawk-Dove and Producer-Scrounger scenarios. Since rewards tend to be of smaller magnitude for the Resource Exploitation scenario, we have taken the smaller value $J = 0.0025$ for this scenario (we could have also just doubled all rewards).

Learning outcomes

Figure 2(a)-(c) of the main text illustrate the results of learning with no inflation bias for the Hawk-Dove scenario. Figures S1(a)-(c) and S2(a)-(c) illustrate learning with no inflation bias in the other two scenarios. Figure 2(d) of the main text and Figures S1(d) and S2(d) show how the payoff to a mutant depends on its inflation bias when resident population members have unbiased subjective rewards (inflation bias $\alpha = 1$).

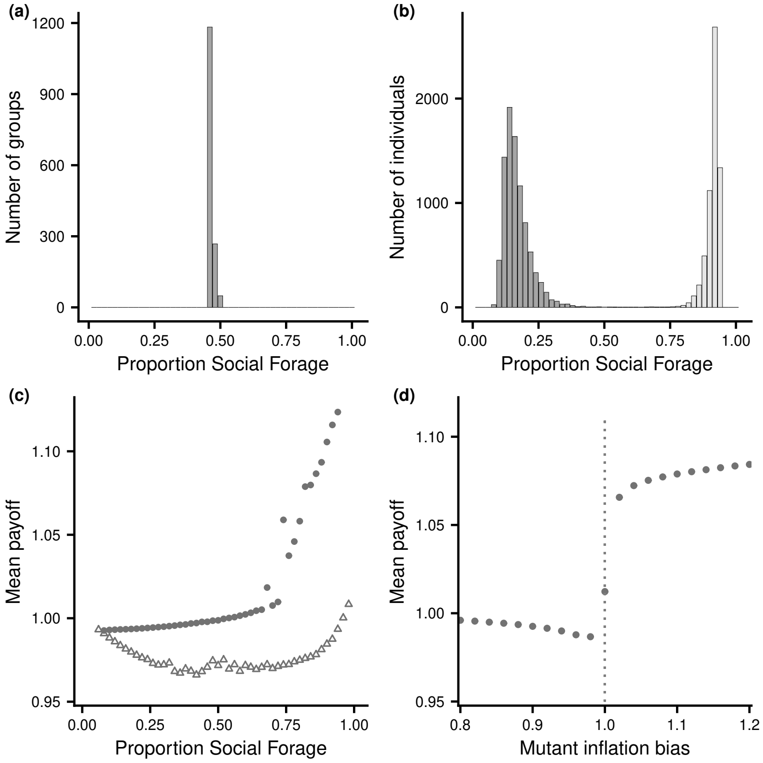

Figure S1. Learning outcomes for the Resource Exploitation game. A group of 15000 individuals is divided into 1500 groups of size $G = 10$. Members of each group play repeated rounds of the game against one another, using the simple learning rule. (a) The number of groups where the overall proportion of choices that are to forage socially made by members of the group take a given value. (b) The number of individuals that forage socially a given proportion of the time, subdivided into those that have a greater payoff per round when foraging solitarily (dark grey) and those that have a greater payoff per round when foraging socially (light grey). (c) The mean payoff of individuals that forage socially a given proportion of the time: when the inflation bias is $\alpha = 1$ (filled circles) and after the evolution of inflation bias (open triangles). (d) The mean payoff to a mutant with given inflation bias α when all other population members use unbiased subjective rewards ($\alpha = 1$), with each point estimated from 100000 independent simulations of group learning. Parameter value: $V = 5$.

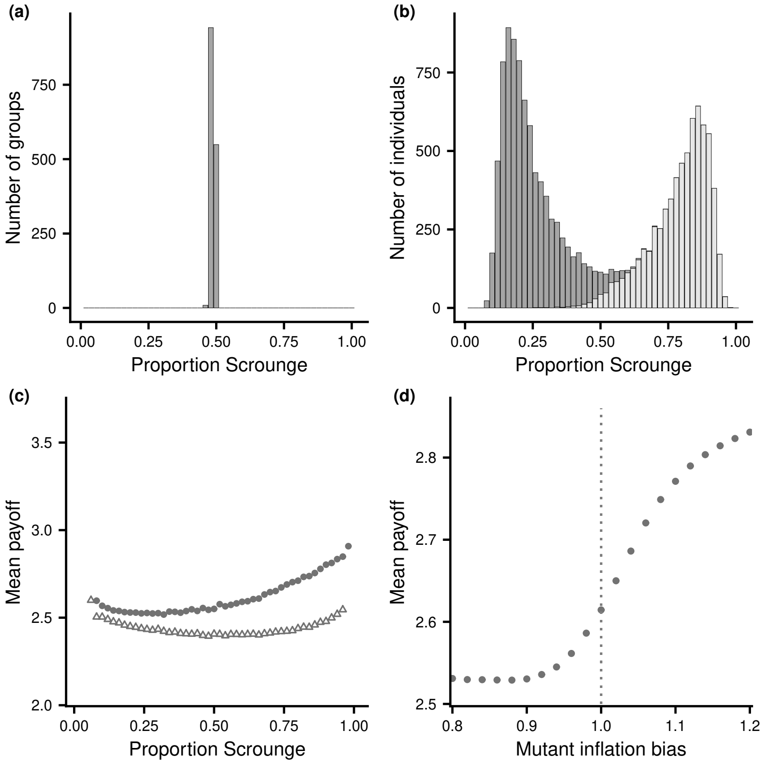

Figure S2. Learning outcomes for the Producer-Scrounger game. A group of 15000 individuals is divided into 1500 groups of size $G = 10$. Members of each group play repeated rounds of the game against one another, using the simple learning rule. (a) The number of groups where the overall proportion of choices that are to scrounge made by members of the group take a given value. (b) The number of individuals that scrounge a given proportion of the time, subdivided into those that have a greater payoff per round when producing (dark grey) and those that have greater payoff per round when scrounging (light grey). (c) The mean payoff of individuals that scrounge a given proportion of the time: when the inflation bias is $\alpha = 1$ (filled circles) and after the evolution of inflation bias (open triangles). (d) The mean payoff to a mutant with given inflation bias α when all other population members use unbiased subjective rewards ($\alpha = 1$), with each point estimated from 100000 independent simulations of group learning. Parameter values: $a = 2, A = 3$.

Appendix C. Evolutionary simulations

Let $\bar{R}$ denote the mean payoff rate during learning. In the Hawk-Dove and the Resource Exploitation scenarios this rate is the total payoff obtained by an individual divided by the total number of rounds played. In the Producer-Scrounger scenario the rate is the total payoff divided by the total time. Note that these payoffs are true fitness increments rather than inflated values. The fitness of an individual is $W = 1 + \bar{R}$, where the background contribution of 1 is the same for all population members.

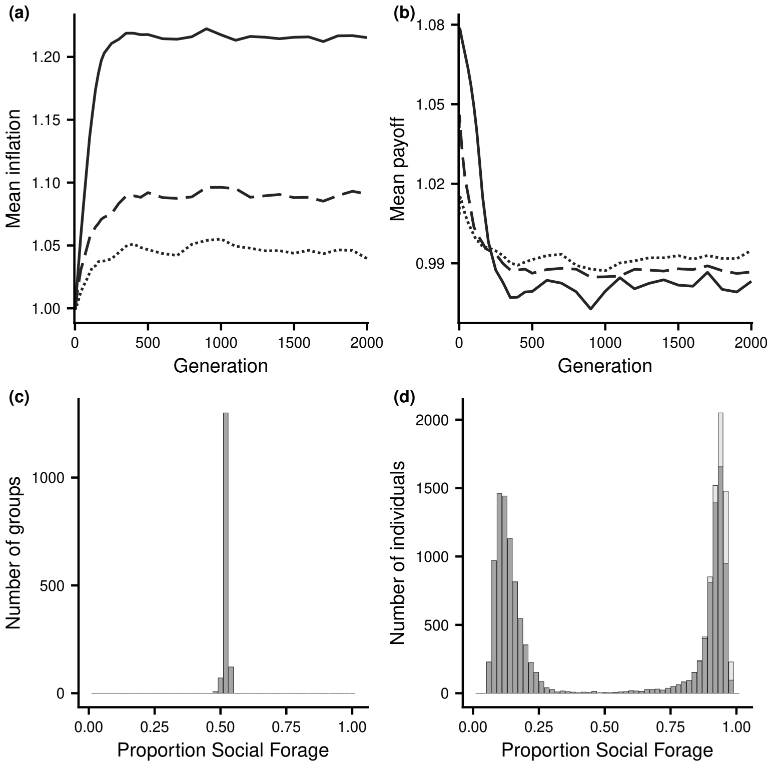

Figure S3. Evolution of the inflation bias for the Resource Exploitation game. (a) Evolved mean bias. (b) The mean payoff of population members. (c) and (d) are the analogues, after the evolution of inflation bias, of panels (a) and (b) respectively in Figure S1. In (a) and (b), group sizes are: $G = 5$ (solid curves), $G = 10$ (dashed curve) and $G = 20$ (dotted curve). In (c) and (d), $G = 10$. Total social reward: $V = 0.5G$. For other details see text.

We performed evolutionary simulations for a population with discrete non-overlapping generations using W as the fitness measure. The evolving trait α is regarded as a quantitative trait. There is a single mating type, but inheritance is sexual in that each individual in the next generation is the offspring of two parents from the current generation. Each parent is chosen with a probability that is proportional to their fitness measure W, with the two choices being independent. Inheritance is specified by the infinitesimal model: the trait of the offspring is the average trait of the two parents plus an error that is normally distributed with mean zero and standard deviation σ. All computations are based on the value $\sigma = 0.02$. For the properties and merits of this form of inheritance see [1]. In all simulations $\alpha = 1$ for all population members in generation 0. The population is size $N = 15000$ in this and all subsequent generations.

Figure 4 of the main text illustrates the evolution of α and the resultant changes in population characteristics for the Hawk-Dove scenario. Figures S3 and S4 illustrate the

analogous evolutionary simulations for the Resource Exploitation game and the Producer-Scrounger game, respectively.

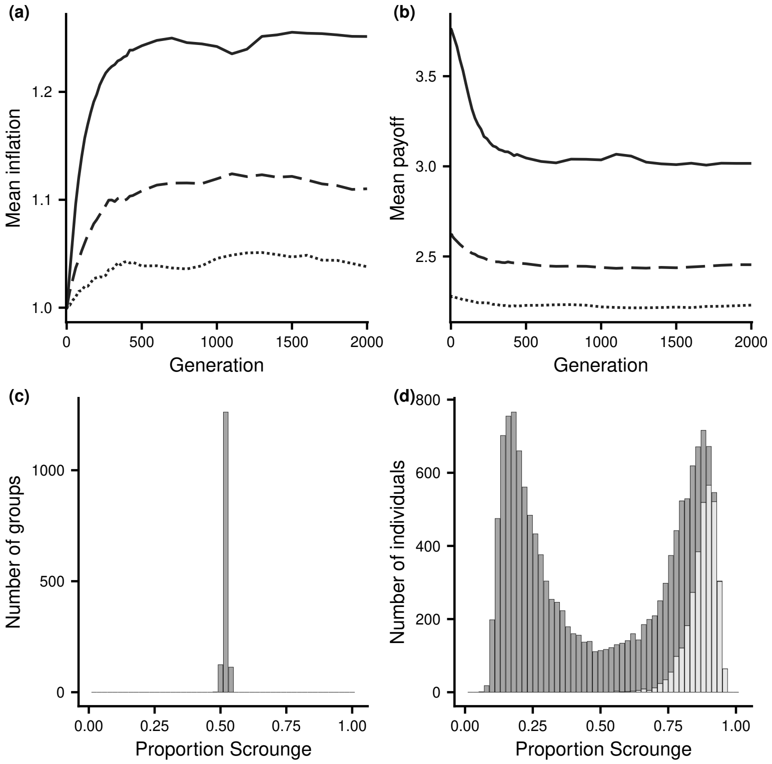

Figure S4. Evolution of the inflation bias for the Producer-Scrounger game. (a) Evolved mean bias. (b) The mean payoff of population members. (c) and (d) are the analogues, after the evolution of inflation bias, of panels (a) and (b) respectively in Figure S2. In (a) and (b), group sizes are: $G = 5$ (solid curves), $G = 10$ (dashed curve) and $G = 20$ (dotted curve). In (c) and (d), $G = 10$. Parameters $a = 2, A = 3$. For other details see text.

References

1. Barton NH, Etheridge AM, Véber A. The infinitesimal model: Definition, derivation, and implications. Theoretical Population Biology. 2017;118:50–73.
